# Active Components of 16 Essential Oils and Their Fumigation Effects on *Galleria mellonella* (Lepidoptera: Pyralidae)

**DOI:** 10.3390/insects15120977

**Published:** 2024-12-09

**Authors:** Xiao-Ling Su, Zhi-Chu Huang, Lin Chen, Dao-Yin Chen, Dong-Xu Zhao, Zhi-Jiang Zeng

**Affiliations:** 1 Honeybee Research Institute, Jiangxi Agricultural University, Nanchang 330045, China; jhmfyjs@163.com; 2 Jinhua Academy of Agricultural Sciences, Jinhua 321017, China; zhichu@zju.edu.cn (Z.-C.H.); daoyin-chen@outlook.com (D.-Y.C.); 15067089856@163.com (D.-X.Z.); 3 Lishui Institute of Agricultural and Forestry Sciences, Lishui 323000, China; fay321@126.com; 4 Jiangxi Province Key Laboratory of Honeybee Biology and Beekeeping, Nanchang 330045, China

**Keywords:** essential oil, *Galleria mellonella*, wax moth eggs, fifth instar larvae, fumigation toxicity

## Abstract

The greater wax moth (GWM, *Galleria mellonella*) is a prevalent pest of the honeybee and a significant risk to both honeybee populations and honeycomb storage. Research on the toxicity of essential oils (EOs) to GWM larvae has provided promising results, although their ovicidal effects and active ingredients require further study. Identifying effective plant compounds is essential for developing insecticides for GWM control. This study assessed the fumigation efficacy of 16 EOs on GWM eggs and fifth instar larvae and determined the effectiveness of these EOs and their primary components for fumigating fifth larvae. Wintergreen, star anise, and clove oils demonstrated significant insecticidal effects on GWM eggs and fifth instar larvae, resulting in a mortality rate exceeding 80% within 48 h. Gas chromatography-mass spectrometry analysis identified methyl salicylate (93.26%), *trans*-anethole (87.75%), and eugenol (77.75%) as the primary compounds in wintergreen, star anise, and clove oils, respectively. Further toxicity testing confirmed that these compounds were responsible for the observed insecticidal properties of the EOs. Notably, *trans*-anethole exhibited the lowest LC_50_ value (25.22 μL/L) against the fifth instar larvae of GWM and significant toxicity against GWM eggs and fifth instar larvae, suggesting its potential as a viable option for the future control of GWM populations.

## Simple Summary

This study aimed to evaluate the active constituents and fumigation effectiveness of 16 distinct plant essential oils (EOs) on greater wax moth (GWM) eggs and fifth instar larvae. The results show that wintergreen, star anise, and clove oils exerted significant insecticidal effects on both GWM eggs and fifth instar larvae. Methyl salicylate and *trans*-anethole, which constitute 93.26% and 87.75% of wintergreen and star anise oils, respectively, function as the sole active ingredient of their respective oils. Eugenol, which constitutes 77.75% of clove oil, is primarily responsible for its insecticidal activity, although it is not the sole contributor. All these EO components exhibit significant insecticidal properties, with *trans*-anethole demonstrating the highest level of toxicity. Our research adds valuable insights to the literature by presenting the first investigation into the effectiveness of EOs for fumigating GWM eggs. Moreover, the results highlight the potential use of anise oil as a novel approach for managing GWM, thus offering a more efficient alternative to traditional methods.

## 1. Introduction

As important pollinating insects, honeybees play an important role in the biodiversity of ecosystems and sustainable development of agriculture [1,2]. Unfortunately, changes in the environment and land use and decreases in bee health have led to a decline in honeybee populations worldwide [3,4,5]. With the development of apiculture, the pest control of honeybees has become a topic of concern. One significant threat to honeybees is the greater wax moth (GWM, *Galleria mellonella*), a major pest of honeybees (especially *Apis cerana*) worldwide [5]. It causes damage to the honeycomb and bee health and often leads to the loss of *A. cerana* colonies [6,7].

Thus, the damage caused by GWM infestations to honeybee colonies and honeycomb storage must be controlled [8]. However, traditional control measures become ineffective in controlling GWM because of the special foraging behaviour of these insects, which includes invasion and burrowing [9]. More importantly, as GWM is a parasite of honeybee hives, chemical control measures must ensure that the health of the bee is not affected, thus increasing the difficulty of GWM control [10]. Currently, chemical fumigation is the preferred practice for controlling insect pests in stored products [11,12], including honeycomb storage without honeybees. However, traditional and efficient fumigation methods, such as using aluminium phosphide [13] and *p*-dichlorobenzene [14,15], present numerous challenges, including host toxicity, residue contamination of honeybee products, drug resistance, and environmental harm. The use of insecticides is subject to increasingly stringent regulations, exemplified by the prohibition of *p*-dichlorobenzene in the European Union, United States, and Canada [6]. Carbon dioxide (CO_2_) fumigation has been proposed as a non-polluting and feasible method for controlling the greater wax moth [7,16]. However, CO_2_ fumigation requires specialised gas storage and transportation infrastructure, as well as a well-sealed preservation environment, which may not be cost-effective for small-scale beekeeping operations [17]. Consequently, there is a critical need to develop safe, economical, and efficient alternatives to controlling GWM.

Plant essential oils (EOs) are valuable natural insecticides for pest control [18,19]. Previous studies have demonstrated the efficacy of some EOs as fumigants because of their ability to repel and induce toxicity in GWM larvae under laboratory conditions. These oils have been shown to inhibit larval and pupal development, potentially leading to abnormalities in GWM development post-larval hatching [20,21,22]. EOs can quickly disperse into the surrounding environment via fumigation and penetrate pests owing to their high volatility and lipophilic properties [23]. Absorption of EOs via the body surface or respiratory system of pests primarily induces toxic effects on the nervous and endocrine systems [24]. Egg and pupal stages are typically the most tolerant to fumigants in many species [25]. However, the fumigation toxicity of EOs against GMW eggs is not known. In addition, the effectiveness of EOs depends on their main components and concentrations, which can vary due to factors such as the season, location, harvest time, and extraction method [26]. Therefore, after determining the effects of EOs, the individual ingredients of these oils must be identified and analysed to develop a stable pest control method.

This study aimed to evaluate the fumigation effectiveness of 16 EOs on the eggs and fifth instar larvae of GWM. EOs exhibiting toxicity towards eggs and larvae were identified along with the active constituents of these oils. Subsequent research was conducted to assess the fumigation toxicity of the EOs that showed high efficacy against fifth instar larvae and identify individual components. This study introduced a promising method for the targeted protection of honeycombs against GWM, thereby paving the way for the development of fumigation strategies that utilise plant-derived compounds.

## 2. Materials and Methods

### 2.1. Insects

The GWM population used in the experiments was obtained from the laboratory of the Jinhua Academy of Agricultural Sciences. This population underwent over 10 generations of breeding and cultivation and was fed aged bee comb material and bee pollen. The larvae of GWM were reared at 30 °C and 60% relative humidity under constant darkness.

### 2.2. EOs and Pure Compounds

Based on the literature, EOs that have been reported to possess insecticidal activity were selected. The EOs used in this study are listed in Table 1. Wintergreen, star anise, clove, tea tree, schizonepeta, perilla, cinnamon, citronella, eucalyptus, peppermint, oriental arborvitae, artemisia, pepper, neem, camphor, and oregano oils were supplied by Huashuo Essential Oil Enterprise stores, China, and prepared by the steam distillation method.

Pure compounds of *trans*-anethole (99%) and eugenol (99%) were purchased from Shanghai McLean Biochemical Technology Co., Ltd. (Shanghai, China), and methyl salicylate (99.9%) was purchased from ANPEL-RTACE Standard Technical Service Co., Ltd. (Shanghai, China).

### 2.3. Fumigation Toxicity Bioassays of GWM Eggs

**Figure 1:**
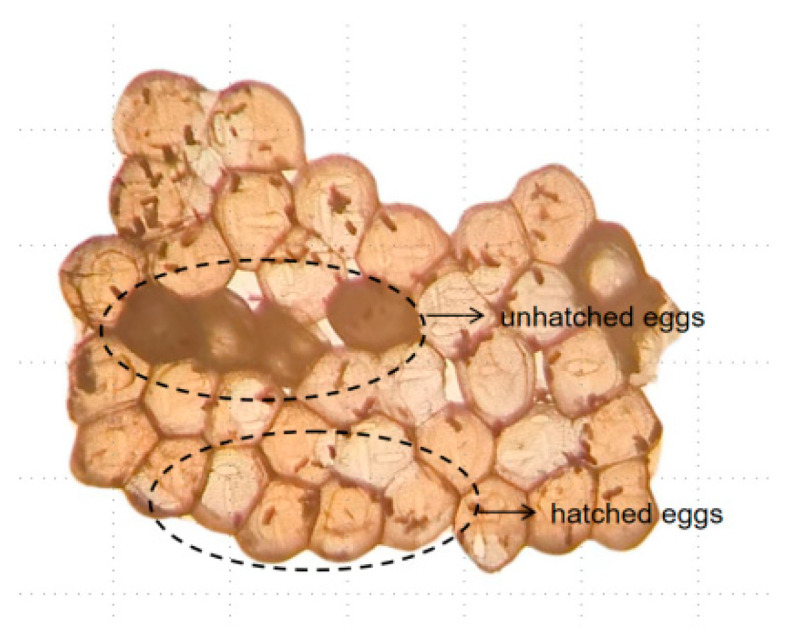
Morphology of the eggs.

The methodology utilised in this study involved EO fumigation assays following the airtight fumigation assay protocol described by Pavela [27] and Jin [28]. Specifically, 50–100 eggs were transferred to a 250 mL Erlenmeyer flask with a rubber plug to ensure a tight seal. Then, a 1 × 5 cm filter paper strip was affixed to the rubber plug using insect needles to ensure that the strip was suspended within the bottle and not in contact with its walls. Subsequently, 40 µL of plant EO (fumigation concentration of approximately 133.33 µL/L) was applied to the filter paper strip. The rubber plug was promptly inserted into the bottle, which was placed in an incubator set at 30 °C, 60% relative humidity, and total darkness for a duration of 48 h. The rubber plug was opened every 24 h to allow for the influx of fresh air. A blank control group was also established. Six replicates were used for each group. Following a 48 h fumigation period, the filter paper strip containing the EO was retrieved. Subsequently, all eggs were allowed to hatch over an 8 d period, after which photographs were captured using a light microscope (K402 McOddy microscope, Motic China Group Co., Ltd., Xiamen, China). The total number of eggs and unhatched eggs (Figure 1) and corrected mortality rates were then determined.

### 2.4. Fumigation Toxicity Bioassay of 5th Instar Larvae

The test method was the same as above. Approximately 40 µL of EOs, with a fumigation concentration of approximately 133.33 µL/L, was administered for the fumigation treatment. The blank control was untreated. Each replicate consisted of ten insects, with six replicates prepared for each group. Throughout the experiment, larvae were not provided any feed. The mortality and corrected mortality of the larvae were calculated.

### 2.5. Gas Chromatography–Mass Spectrometry (GC–MS) Analysis of EOs

The percentage composition of EOs with a mortality rate exceeding 80% was determined using gas chromatography–flame ionizing detection (GC–FID), and the compounds were identified by GC–MS. Prior to analysis, the EO sample (10 μL) was dissolved in 1 mL of hexane, filtered with a 0.22 μm organic filter membrane, and introduced into the instrument. The GC analyses were performed using a Shimadzu GC-2014C (Shimadzu Corp., Kyoto, Japan) equipped with an FID and an Rtx^®^-1 capillary column (25 m × 0.32 mm × 0.5 μm film; Restek, Bellefonte, PA, USA). The oven initial temperature was 35 °C for 3 min, which was increased at a rate of 10 °C/min to 280 °C and then maintained isothermally for 10 min. The temperatures of the injector and detector were 280 °C and 300 °C, respectively. The split ratio was 50:1, with an injection volume of 1 μL. Helium gas was used as the carrier gas at a flow rate of 3 mL/min. Samples were injected in triplicate for quantification.

The chemical characterization of EO compounds was performed using a GC–MS system (7890B-5977B, Agilent Technologies Co., Ltd., Santa Clara, CA, USA) equipped with a HP-5MS chromatographic column (30 m × 250 μm × 0.25 μm). Helium gas was used as the carrier gas at a flow rate of 1 mL/min. The injection volume was 1 μL with a split ratio of 20:1. The injector temperature was set at 280 °C. The temperature program consisted of an initial column temperature of 40 °C for 4 min, which was increased to 280 °C at a rate of 10 °C/min and then maintained for 5 min. The mass spectrometry conditions were as follows: EI mode: ionization energy: 70 eV; ionization source temperature: 230 °C; scan range: 50–550 amu; and scan rate: 200 spectra/s.

The GC–MS data were acquired using MassHunter Workstation Qualitative Analysis Software (Version B.08.00) (Agilent, Santa Clara, CA, USA). The retention indices (RIs) were calculated based on the data of C7–C40 n-alkanes acquired with the same GC–MS method. Identification of the EO components was performed using the NIST17 mass spectral library database by comparing RI values with published data [29,30,31].

### 2.6. Fumigation Toxicity Bioassays of EO Components

The fumigation effects of EO components on GWM eggs and larvae were tested following the methodology described in Section 2.3 and Section 2.4. Six replicates were prepared for each group.

### 2.7. Determination of the LC_50_ for 5th Instar Larvae

The study employed the airtight fumigation assay method outlined above to determine the LC_50_ values of EOs and predominant compounds that demonstrated significant effects. Fifth instar larvae were exposed to different concentrations of the test substances (0 μL/L, 8.33 μL/L, 16.67 μL/L, 33.33 μL/L, 66.67 μL/L, 133.33 μL/L, and 266.67 μL/L), and their mortality rates were assessed after 24 and 48 h. Six replicates were used for each condition.

### 2.8. Statistical Analysis

The corrected mortality was calculated as follows: corrected mortality % = (% of mortality in treatment  −  % of mortality in control)/(100  −  % of mortality in control) [32].

Statistical analyses were performed based on one-way analysis of variance in SPSS version 23 (IBM SPSS Statistics 23) (IBM Corp., Armonk, NY, USA). The regression equation for the relationship between virulence and lethal medium concentration (LC_50_) via fumigation poisoning was determined by converting the corrected death rate to a probability value (y) using the log-probability method and representing the concentration as a base 10 logarithm (x).

## 3. Results

### 3.1. Fumigation Activity

**Figure 2:**
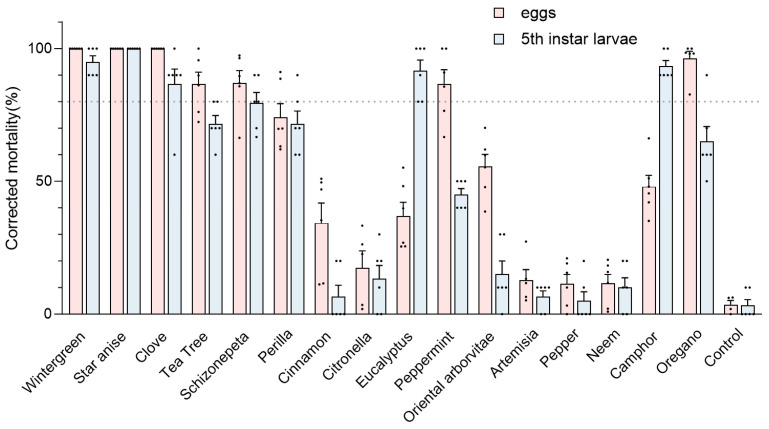
Corrected mortality (%) of greater wax moth (GWM) after essential oil treatment (48 h at 30 °C) based on the fumigation toxicity bioassay. The insects were tested in six replicates (*n* = 10 larvae and *n* = 50–100 eggs each) per formulation. Control value represents the mortality rate of the blank control group.

The fumigation toxicity of 16 EOs against the GWM eggs and fifth instar larvae is reported in Figure 2. The average egg and larvae mortality rates of the control group were very low (3.58% and 3.33%, respectively), indicating the feasibility of this approach. At a concentration of 133.33 μL/L, star anise oil exhibited the highest efficacy, achieving a corrected mortality rate of 100% for both eggs and fifth instar larvae. This was followed by wintergreen oil and clove oil, which also demonstrated a 100% fumigation mortality rate for eggs and over 80% for fifth instar larvae. In contrast, the insecticidal effects of cinnamon, citronella, artemisia, pepper, and neem oils were less than 40%. Interestingly, most EOs displayed greater toxicity towards eggs; however, camphor and eucalyptus oil showed significant insecticidal activity against fifth instar larvae, with mortality rates of 93.15% and 89.72%, respectively.

### 3.2. Chemical Composition of EOs

**Figure 3:**
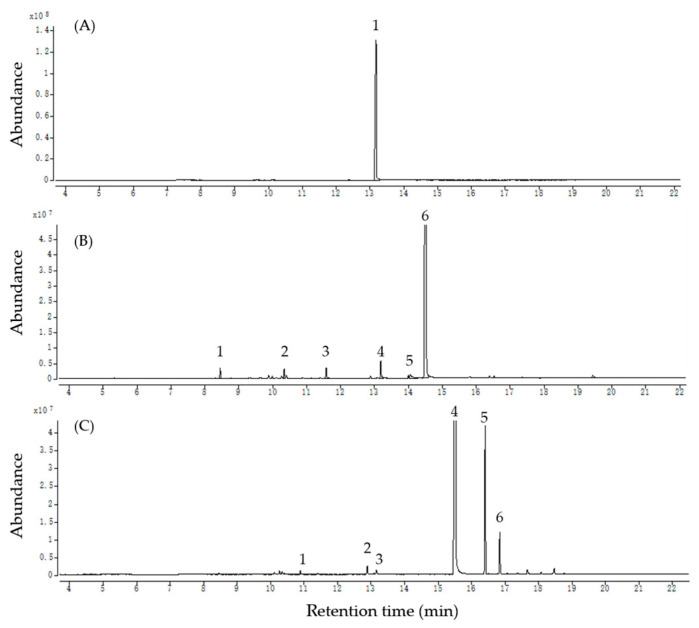
Total ion chromatograms of the three EOs, (**A**) wintergreen oil, (**B**) star anise oil, and (**C**) clove oil.

GC–MS analyses were conducted on the three most active oils, and the components with relative peak areas exceeding 0.5% are listed in Table 2. Total ion chromatograms of the three Eos, (A) wintergreen oil, (B) star anise oil, and (C) clove oil, are reported in Figure 3. The findings reveal that terpenoids were the predominant components of the three EOs. Wintergreen oil consisted solely of methyl salicylate (93.26%). The predominant compound in star anise oil was *trans*-anethole (87.75%), although it also contained α-pinene (1.19%), limonene (1.44%), linalool (1.12%), estragole (1.41%) and *cis*-anethole (0.66%). Clove oil primarily consisted of eugenol (77.75%) and caryophyllene (8.35%) but also included γ-terpinene (0.51%), terpinen-4-ol (0.63%), methyl salicylate (0.75%), and humulene (2.24%).

### 3.3. Fumigation Activity of EO Components on Eggs and Larvae

**Figure 4:**
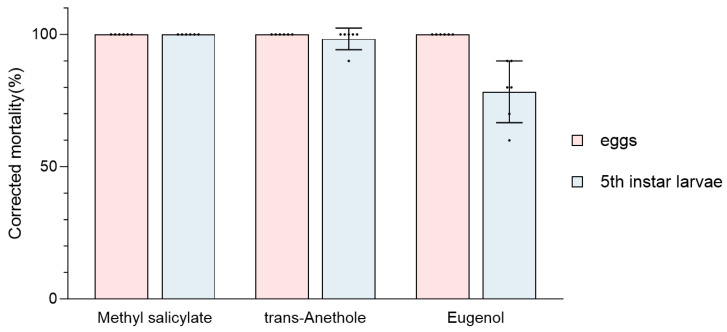
Corrected mortality rate (%) of essential oil components on the eggs and 5th instar larvae of GWM. The insects were tested based on six replicates (*n* = 10 larvae and *n* = 50–100 eggs each) per formulation.

The fumigation efficacy of the primary components methyl salicylate, *trans*-anethole, and eugenol on GWM eggs and fifth instar larvae was investigated (Figure 4). The three components resulted in 100% mortality rates for GWM eggs. Furthermore, after 48 h of fumigation, the corrected mortality rates for fifth instar larvae were 100%, 100%, and 78.33% for methyl salicylate, *trans*-anethole, and eugenol, respectively.

### 3.4. Fumigant Toxicity of EOs and Main Components on Fifth Instar Larvae

Table 3 shows the LC_50_ and LC_90_ values of the EOs and their primary monomer constituents studied against GWM fifth instar larvae after 24 h and 48 h of exposure. As observed, a gradual decrease in the LC_50_ values over time was observed for the three plant EOs. Specifically, at the 24 h time point, the LC_50_ levels were ranked in the following order from lowest to highest: wintergreen at 51.07 μL/L, star anise at 64.61 μL/L, and clove oil at 91.30 μL/L. Following a 48 h exposure period, clove oil displayed the highest larvicidal activity with an LC_50_ of 29.24 μL/L and LC_90_ of 86.25 μL/L.

The 48 h fumigant toxicity exhibited by methyl salicylate and *trans*-anethole surpassed that of the EOs. In contrast, eugenol displayed lower fumigant toxicity than clove oil. Moreover, *trans*-anethole, which is the main compound of star anise oil, was efficient against larvae, with a 48 h LC_50_ of 25.22 μL/L. Additionally, *trans*-anethole was the compound with the greater slope, indicating that small changes in concentration were required to increase the mortality of GWM.

## 4. Discussion

This study tested the fumigation efficacy of 16 EOs on GWM eggs and fifth instar larvae based on a biological assay. Wintergreen, star anise, clove, oregano, tea tree, and peppermint oils demonstrated a fumigation mortality rate exceeding 80% on GMW eggs at a concentration of 133.33 μL/L. This is the first report on the fumigation efficacy of EOs on GWM eggs; thus, the findings provide important insights into their fumigation effects. Our study also revealed that the EOs of eucalyptus, anise, camphor, and clove had fumigation mortality rates exceeding 80% for fifth instar larvae of GWM (Figure 2).

Interestingly, most EOs displayed greater toxicity towards eggs; however, camphor and eucalyptus oil showed significant insecticidal activity against fifth instar larvae, with mortality rates of 93.15% and 89.72%, respectively. Studies show that plant extracts and EOs are toxic to insect larvae and eggs [33]. Typically, the dense eggshell of insect eggs poses a challenge for the penetration of insecticides, thereby limiting their toxic effects and rendering the eggs more resistant to insecticides compared to the larval stage [34,35]. The methanol extract of *Pisonia alba* effectively prevents and controls the larvae, pupae, and eggs of *Aedes aegypti* and *Culex quinquefasciatus* mosquitoes, with a higher LC_50_ for eggs than third instar larvae [36]. Nevertheless, the efficacy of EOs varies with the developmental stage and species of the insect and the botanical source of the oil. *Ephestia kuehniella* eggs are less tolerant to EOs than their final larvae [37]. *Helicoverpa armigera* eggs are more susceptible to oregano essential oil than third instar larvae, as indicated by the higher LC_50_ [38]. In contrast, these eggs are less sensitive to *Cheilocostus speciosus* EO [39]. In this study, most EOs, except eucalyptus and sassafras, showed stronger fumigation effects on GWM eggs at the same concentrations and exposure times. This may be due to the larger size of the fifth instar larvae used in the study, because older larvae generally require higher concentrations of EOs for effectiveness [40].

Consistent with our results, one study reported the fumigation efficiency of clove oil, eugenol, and methyl salicylate (the primary component of wintergreen oil) on the fifth instar larvae [41]. The potential efficacy of star anise EO has not yet been documented. Nonetheless, previous studies on *Ctenocephalides felis* [42] and *Schistosoma mansoni* [43] have reported the insecticidal effects of anise oil. Our findings present a new application of anise oil as a potential method for controlling GWM, thus providing a more effective EO alternative for mitigating GWM damage and establishing a basis for future research and control strategies.

The insecticidal effects of EOs are mainly due to their main compounds [26,44], although minor components may also play a role in their larvicidal activity [45]. In the present study, methyl salicylate, the primary compound identified in wintergreen oil at a concentration exceeding 93% [46], demonstrated a complete insecticidal effect and showed similar efficacy on GWM eggs and larvae, as well as comparable LC_50_ values. In addition, *trans*-anethole (87.75%) and eugenol (77.75%) represented the primary constituents of star anise and clove oil, respectively, and their primary constituents were similar to those reported previously [47,48]. The results show that *trans*-anethole had a lower LC_50_ than star anise oil, indicating it is the only active ingredient. This could be why a higher dose of star anise oil is required for the same insecticidal effect, potentially due to other compounds masking this distinction. These findings align with previous research by Pavela et al. [49] on *Culex quinquefasciatus* larvae. Contrary to the above findings, the LC_50_ value of eugenol was higher than that of clove oil, which was consistent with the trend in the study by Owayss [41]. Other compounds in clove oil, particularly caryophyllene (8.35%), may contribute significantly to its insecticidal effectiveness. Caryophyllene displays various biological activities, such as antibacterial and insecticidal activity [50,51], underscoring the need for additional research to explore its potential insecticidal effects. Nonetheless, the insecticidal efficacy of eugenol remained satisfactory because the 48 h toxicity levels of eugenol surpassed those of star anise and wintergreen oils.

The highest fumigation toxicity towards the fifth instar larva of GWM over 48 h was observed for *trans*-anethole relative to the other substances evaluated. Consequently, using this particular compound may allow for a reduction in drug dosage without compromising the insecticidal efficacy, thereby potentially mitigating adverse odour profiles and medication costs. Moreover, *trans*-anethole is a predominant compound in various EOs, such as dill (*Foeniculum vulgare* Mill.), anise (*Pimpinella anisum* L.), and star anise (*Illicium verum* Hook.f.), and it exhibits significant fumigation potential [52,53,54,55]. The effects of these EOs vary depending on the concentration of *trans*-anethole present, and the utilisation of constituents in their pure form does not exhibit this variability [56]. In addition to the insecticidal properties against pests, *trans*-anethole has been shown to exhibit broad antifungal activity against the budding yeast *Saccharomyces cerevisiae* [57], plant pathogenic fungi [58], and *Enterococcus faecalis* [59]. We speculate that it plays a positive role in the disinfection of honeycombs, although this requires further study.

In terms of safety, methyl salicylate, *trans*-anethole, and eugenol are safe and non-toxic, making them suitable for application in the food industry as preservatives and additives. However, the use of EOs can alter the flavour profile of honey. EO residues such as thymol present in honey are significantly below the recommended safety threshold and diminish rapidly due to their volatile nature, suggesting that there may be no associated food safety risk for consumers [60]. Additionally, these substances are readily accessible and economically viable.

In the apiary industry, preparations based on EOs have been approved for the control of parasitic bee mites [61]. Natural pesticide products containing eugenol, such as EcoPCOR, have also been commercialised for the control of crawling and flying insects [62]. In practical production, the comb can be preserved when using EOs by placing the combs into multi-layer hives. A specified quantity of EO is applied directly to an appropriate carrier, and then the hive is sealed with plastic film to ensure airtight storage. Nevertheless, this study solely examined EOs and their constituents in a controlled laboratory setting. External factors such as fluctuations in ambient temperature and the integrity of container seals could potentially impact the effectiveness of EOs in practical applications. Future research should explore the impact of EOs on GWM under field conditions and develop formulations with enhanced efficacy, stability, and cost-effectiveness. These studies could provide valuable insights into the application of EOs as novel insecticides/fumigants, thereby contributing to the development of targeted and effective methods for controlling specific pests.

## 5. Conclusions

This study revealed that wintergreen, star anise, and clove oils exhibited notable fumigation effects on the eggs and fifth instar larvae of GWM through bioassay testing. Moreover, methyl salicylate, *trans*-anethole, and eugenol were identified as the active fumigant components in these oils. Among them, *trans*-anethole showed the strongest insecticidal activity against GWM. This suggests that these components could work well as fumigants for bee combs to stop moth infestations, thereby offering a natural management option.

## Figures and Tables

**Table 1:** Plant species and essential oils tested in this study.

Essential Oil	Family	Scientific Name	Extracted Plant Part
Wintergreen	Aquifoliaceae	*Ilex purpurea*	leaf
Star anise	Magnoliaceae	*Illicium verum*	seed
Clove	Myrtaceae	*Syzygium aromaticum*	flower bud
Tea tree	Myrtaceae	*Melaleuca alternifolia*	leaf
Schizonepeta	Lamiaceae	*Nepeta cataria*	leaf
Perilla	Lamiaceae	*Perilla frutescens*	leaf
Cinnamon	Lauraceae	*Cinnamomum cassia*	leaf, bark
Citronella	Poaceae	*Cymbopogon citratus*	leaf
Eucalyptus	Myrtaceae	*Eucalyptus globulus*	leaf
Peppermint	Lamiaceae	*Mentha piperita*	leaf
Arborvitae	Cupressaceae	*Platycladus orientalis*	leaf
Artemisia	Asteraceae	*Artemisia argyi*	stem, leaf
Pepper	Rutaceae	*Zanthoxylum bungeanum*	seed
Neem	Meliaceae	*Melia azedarach*	seed
Camphor	Lauraceae	*Camphora parthenoxylon*	wood
Oregano	Lamiaceae	*Origanum vulgare*	leaf

**Table 2:** Analysis of the main chemical components of the essential oils (EOs).

EOs	No.	RI_exp_	RI_lit_	Chemical Compounds	Relative Content (%)
Wintergreen	1	1196	1195	Methyl salicylate	93.26 ± 0.38
Star anise	1	936	938	α-Pinene	1.19 ± 0.01
	2	1032	1031	Limonene	1.44 ± 0.02
	3	1102	1101	Linalool	1.12 ± 0.01
	4	1204	1202	Estragole	1.41 ± 0.01
	5	1259	1259	*cis*-Anethole	0.66 ± 0.02
	6	1295	1289	*trans*-Anethole	87.75 ± 0.47
Clove	1	1056	1062	γ-Terpinene	0.51 ± 0.02
	2	1177	1175	Terpinen-4-ol	0.63 ± 0.01
	3	1193	1195	Methyl salicylate	0.75 ± 0.02
	4	1359	1360	Eugenol	77.75 ± 0.21
	5	1422	1419	Caryophyllene	8.35 ± 0.1
	6	1457	1452	Humulene	2.25 ± 0.04

Abbreviations: RI_exp_: experimental retention index; RI_lit_: literature data.

**Table 3:** Fumigation toxicity of essential oil constituents (EOCs) on 5th instar larvae of GWM.

EOCs	Time (h)	LC_50_ (95% CI) ^a^ (μL/L)	LC_90_ (95% CI) ^a^ (μL/L)	Slope ± SE ^b^	χ^2 c^	*p*-Value
Wintergreen oil	24	51.07 (41.98–61.40)	139.51 (107.69–210.26)	2.94 ± 0.30	43.19	<0.001
	48	39.60 (32.74–46.72)	92.06 (75.29–122.93)	3.50 ± 0.32	48.64	<0.001
Star anise oil	24	64.61 (49.46–86.40)	246.85 (157.97–619.46)	2.02 ± 0.29	47.08	<0.001
	48	42.18 (33.07–53.78)	107.98 (82–174.60)	3.22 ± 0.34	65.49	<0.001
Clove oil	24	91.30 (71.70–128.25)	405.29 (239.78–1182.32)	1.98 ± 0.30	30.09	<0.001
	48	29.24 (21.53–36.63)	86.25 (66.53—129.99)	2.73 ± 0.30	53.34	<0.001
Methyl salicylate	24	42.68 (35.45–50.79)	125.36 (97.11–185.88)	2.74 ± 0.36	6.21	<0.001
	48	32.87 (26.56–39.30)	98.11 (76.80–143.44)	2.70 ± 0.37	4.87	<0.001
*trans*-Anethole	24	45.29 (29.59–63.66)	702.381 (349.47–2794.64)	1.08 ± 0.19	28.84	<0.001
	48	25.22 (19.21–31.07)	64.68 (50.46–96.92)	3.13 ± 0.41	33.71	<0.001
Eugenol	24	249.08 (173.59–455.34)	2023.33 (904.44–9394.68)	1.41 ± 0.23	16.75	<0.001
	48	36.41 (28.16–45.16)	204.83 (149.01–327.29)	1.71 ± 0.20	29.59	<0.001

Abbreviations: ^a^: lethal concentration (μL/L) to 50% (LC_50_) or 90% (LC_90_) of the exposed population; CI, confidence limit; ^b^: SE, standard error; ^c^: χ^2^, chi-square.

## Data Availability

The data presented in this study are available in this article.

## References

[1] Gallai N., Salles J.M., Settele J., Vaissière B.E. (2009). Economic valuation of the vulnerability of world agriculture confronted with pollinator decline. Ecol. Econ..

[2] van der Sluijs J.P., Vaage N.S. (2016). Pollinators and global food security: The need for holistic global stewardship. Food Ethics.

[3] Potts S.G., Biesmeijer J.C., Kremen C., Neumann P., Schweiger O., Kunin W.E. (2010). Global pollinator declines: Trends, impacts and drivers. Trends Ecol. Evol..

[4] Kleijn D., Kohler F., Báldi A., Batáry P., Concepción E.D., Clough Y., Díaz M., Gabriel D., Holzschuh A., Knop E. (2009). On the relationship between farmland biodiversity and land-use intensity in Europe. Proc. R. Soc. B Biol. Sci..

[5] Yordanova M., Evison S.E.F., Gill R.J., Graystock P. (2022). The threat of pesticide and disease co-exposure to managed and wild bee larvae. Int. J. Parasitol. Parasites Wildl..

[6] Kwadha C.A., Ong’amo G.O., Ndegwa P.N., Raina S.K., Fombong A.T. (2017). The biology and control of the greater wax moth, *Galleria mellonella*. Insects.

[7] Gulati R., Kaushik H.D. (2004). Enemies of honeybees and their management—A review. Agric. Rev..

[8] Ellis J.D., Graham J.R., Mortensen A. (2013). Standard methods for wax moth research. J. Apicult Res..

[9] Nielsen R.A., Brister C. (1979). Greater wax moth: Behavior of larvae. Ann. Entomol. Soc. Am..

[10] (2010). Rosenkranz P, Aumeier P, Ziegelmann B, Biology and control of *Varroa destructor*. J. Invertebr. Pathol..

[11] Sousa A.H., Faroni L.R.D., Pimentel M.A.G., Guedes R.N.C. (2009). Developmental and population growth rates of phosphine-resistant and susceptible populations of stored-product insect pests. J. Stored Prod. Res..

[12] Feng S., Opit G., Deng W., Stejskal V., Li Z. (2022). A chromosome-level genome of the booklouse, *Liposcelis brunnea*, provides insight into louse evolution and environmental stress adaptation. Gigascience.

[13] Rajendran S., Hajira Parveen K.M. (2005). Insect infestation in stored animal products. J. Stored Prod. Res..

[14] Bogdanov S., Kilchenmann V., Seiler K., Pfefferli H., Frey T., Roux B., Wenk P., Noser J. (2004). Residues of para-dichlorobenzene in honey and beeswax. J. Apicult Res..

[15] Tananaki C., Thrasyvoulou A., Karazafiris E., Zotou A. (2006). Contamination of honey by chemicals applied to protect honeybee combs from wax moth (*Galleria mellonela* L.). Food Addit. Contam..

[16] Shimanuki H., Knox D. (1997). Bee health and international trade. Rev. Sci. Tech..

[17] Donahaye E.J. (2000). Current status of non-residual control methods against stored product pests. Crop Prot..

[18] Robu V., Covaci G., Popescu I.M. (2015). The use of essential oils in organic farming. Res. J. Agric. Sci..

[19] Isman M.B. (2020). Botanical insecticides in the twenty-first century—Fulfilling their promise?. Annu. Rev. Entomol..

[20] Zaitoun S.T. (2007). The effect of different Mediterranean plant extracts on the development of the great wax moth *Galleria mellonella* L. (Lepidoptera: Pyralidae) and their toxicity to worker honeybees *Apis mellifera* L. (Hymenoptera: Apidae) under laboratory conditions. J. Food Agric. Environ..

[21] Elmubarak S.M.E. (2007). The Effect of Water Extract of Neem and Camphor Leaves on the Last Instar of the Greater Wax Moth *Galleria mellonella* L. (Lepidoptera: Pyralidae). Ph.D. Dissertation.

[22] Elbehery H., El-Wahab T.E.A., Dimetry N.Z. (2016). Management of the greater wax moth *Galleria mellonella* with neem Azal-T/S, in the laboratory and under semi-field conditions. J. Apicult Res..

[23] Mossa A.T.H. (2016). Green pesticides: Essential oils as biopesticides in insect-pest management. J. Environ. Sci. Technol..

[24] Rattan R.S. (2010). Mechanism of action of insecticidal secondary metabolites of plant origin. Crop Prot..

[25] Hole B.D., Bell C.H., Mills K.A., Goodship G. (1976). The toxicity of phosphine to all developmental stages of thirteen species of stored product beetles. J. Stored Prod. Res..

[26] Fiocco D., Fiorentino D., Frabboni L., Benvenuti S., Orlandini G., Pellati F., Gallone A. (2011). Lavender and peppermint essential oils as effective mushroom tyrosinase inhibitors: A basic study. Flavour. Fragr. J..

[27] Pavela R., Vrchotova N., Triska J. (2009). Mosquitocidal activities of thyme oils (*Thymus vulgaris* L.) against *Culex quinquefasciatus* (Diptera: Culicidae). Parasitol. Res..

[28] Jin C., Han H., Xie Y., Li B., Zhang Z., Zhang D. (2022). Toxicity, behavioral effects, and chitin structural chemistry of reticulitermes flaviceps exposed to *Cymbopogon citratus* EO and its major constituent citral. Insects.

[29] Zhang W.J., Liu C., Yang R.J., Zheng T.T., Zhao M.M., Ma L., Yan L. (2019). Comparison of volatile profiles and bioactive components of sun-dried Pu-erh tea leaves from ancient tea plants on Bulang Mountain measured by GC-MS and HPLC. J. Zhejiang Univ. Sci. B.

[30] Hlebová M., Foltinová D., Vešelényiová D., Medo J., Šramková Z., Tančinová D., Mrkvová M., Hleba L. (2022). The vapor phase of selected essential oils and their antifungal activity in vitro and in situ against *Penicillium commune*, a common contaminant of cheese. Foods.

[31] Radušienė J., Karpavičienė B., Marksa M., Ivanauskas L., Raudonė L. (2022). Distribution patterns of essential oil terpenes in native and invasive solidago species and their comparative assessment. Plants.

[32] Abbott W.S. (1925). A method of computing the effectiveness of an insecticide. J. Econ. Entomol..

[33] Baz M.M., Selim A.M., Radwan I.T., Alkhaibari A.M., Gattan H.S., Alruhaili M.H., Alasmari S.M., Gad M.E. (2024). Evaluating larvicidal, ovicidal and growth inhibiting activity of five medicinal plant extracts on *Culex pipiens* (Diptera: Culicidae), the West Nile virus vector. Sci. Rep..

[34] Kuppusamy C., Murugan K. (2008). Mosquitocidal effect of *Euphorbia heterophylla* Linn against the Bancroftian filariasis vector, *Culex quinquefasciatus Say* (Diptera: Culicidae). Int. J. Integr. Biol..

[35] Fikru S., Tolossa K., Lindemann P., Bucar F., Asres K. (2024). Larvicidal, ovicidal, and repellent activities of *Leucas stachydiformis* (Hochst. ex Benth.) Briq essential oil against *Anopheles arabiensis*. J. Trop. Med..

[36] Vilvest J., Milton M.C.J., Yagoo A. (2024). Evaluating the effectiveness of *Pisonia alba* leaf extracts in managing *Aedes aegypti* and *Culex quinquefasciatus* populations via larvicidal, pupicidal and ovicidal actions. Acta Parasitol..

[37] Saraç A., Tunç I. (1995). Toxicity of essential oil vapours to stored-product insects. J. Plant Dis. Prot..

[38] Gong X., Ren Y. (2020). Larvicidal and ovicidal activity of carvacrol, p-cymene, and γ-terpinene from *Origanum vulgare* essential oil against the cotton bollworm, *Helicoverpa armigera* (Hübner). Environ. Sci. Pollut. Res. Int..

[39] Benelli G., Govindarajan M., Rajeswary M., Vaseeharan B., Alyahya S.A., Alharbi N.S., Kadaikunnan S., Khaled J.M., Maggi F. (2018). Insecticidal activity of camphene, zerumbone and α-humulene from *Cheilocostus speciosus* rhizome essential oil against the old-world bollworm, *Helicoverpa armigera*. Ecotox Environ. Safe.

[40] Pushpanathan T., Jebanesan A., Govindarajan M. (2006). Larvicidal, ovicidal and repellent activities of *Cymbopogan citratus* Stapf (Graminae) essential oil against the filarial mosquito *Culex quinquefasciatus* (Say) (Diptera: Culicidae). Trop. Biomed..

[41] Owayss A.A., Abd-Elgayed A.A. (2007). Potential efficacy of certain plant volatile oils and chemicals against greater wax moth, *Galleria mellonella* L. (Lepidoptera: Pyralide). Bull. Ent Soc. Egypt. Econ. Ser..

[42] Freitas J.P., De Jesus I.L.R., Chaves J.K.O., Gijsen I.S., Campos D.R., Baptista D.P., Ferreira T.P., Alves M.C.C., Coumendouros K., Cid Y.P. (2021). Efficacy and residual effect of *Illicium verum* (star anise) and *Pelargonium graveolens* (rose geranium) essential oil on cat fleas *Ctenocephalides felis felis*. Rev. Bras. Parasitol. Vet..

[43] Wakabayashi K.A., De Melo N.I., Aguiar D.P., de Oliveira P.F., Groppo M., da Silva Filho A.A., Rodrigues V., Cunha W.R., Tavares D.C., Magalhães L.G. (2015). Anthelmintic effects of the essential oil of fennel (*Foeniculum vulgare* Mill., Apiaceae) against *Schistosoma mansoni*. Chem. Biodivers..

[44] Lahlou M. (2004). Methods to study the phytochemistry and bioactivity of essential oils. Phytother. Res..

[45] Silva W.J., Dória G.A., Maia R.T., Nunes R.S., Carvalho G.A., Blank A.F., Alves P.B., Marçal R.M., Cavalcanti S.C.H. (2008). Effects of essential oils on *Aedes aegypti* larvae: Alternatives to environmentally safe insecticides. Bioresour. Technol..

[46] Ojha P.K., Poudel D.K., Dangol S., Rokaya A., Timsina S., Satyal P., Setzer W.N. (2022). Volatile constituent analysis of wintergreen essential oil and comparison with synthetic methyl salicylate for authentication. Plants.

[47] Khan B.A., Ahmad S., Khan M.K., Hosny K.M., Bukhary D.M., Iqbal H., Murshid S.S., Halwani A.A., Alissa M., Menaa F. (2022). Fabrication and characterizations of pharmaceutical emulgel co-loaded with naproxen-eugenol for improved analgesic and anti-Inflammatory effects. Gels.

[48] Kwiatkowski P., Pruss A., Masiuk H., Mnichowska-Polanowska M., Kaczmarek M., Giedrys-Kalemba S., Sienkiewicz M. (2019). The effect of fennel essential oil and trans-anethole on antibacterial activity of mupirocin against *Staphylococcus aureus* isolated from asymptomatic carriers. Postep. Dermatol. I Alergol..

[49] Pavela R. (2014). Insecticidal properties of Pimpinella anisum essential oils against the *Culex quinquefasciatus* and the non-target organism *Daphnia magna*. J. Asia-Pac. Entomol..

[50] Park M.H., Kim C.J., Lee J.Y., Kim I.S., Kin S. (2021). Development and validation of a gas chromatography method for the determination of β-caryophyllene in clove extract and its application. Sci. Rep..

[51] Govindarajan M., Rajeswary M., Hoti S.L., Bhattacharyya A., Benelli G. (2016). Eugenol, α-pinene and β-caryophyllene from *Plectranthus barbatus* essential oil as eco-friendly larvicides against malaria, dengue and Japanese encephalitis mosquito vectors. Parasitol. Res..

[52] Yeom H.J., Kang J.S., Kim G.H., Park I.K. (2012). Insecticidal and acetylcholine esterase inhibition activity of Apiaceae plant essential oils and their constituents against adults of German cockroach (*Blattella germanica*). J. Agric. Food Chem..

[53] Li S.G., Zhou B.G., Li M.Y., Liu S., Hua R.M., Lin H.F. (2017). Chemical composition of *Illicium verum* fruit extract and its bioactivity against the peach–potato aphid, *Myzus persicae* (Sulzer). Arthropod-Plant Interact..

[54] Tak J.H., Jovel E., Isman M.B. (2016). Contact, fumigant, and cytotoxic activities of thyme and lemongrass essential oils against larvae and an ovarian cell line of the cabbage looper, *Trichoplusia ni*. J. Pest. Sci..

[55] Wang Z., Xie Y., Sabier M., Zhang T., Deng J., Song X., Liao Z., Li Q., Yang S., Cao Y. (2021). Trans-anethole is a potent toxic fumigant that partially inhibits rusty grain beetle (*Cryptolestes ferrugineus*) acetylcholinesterase activity. Ind. Crop Prod..

[56] Kostić I., Lazarević J., Šešlija-Jovanović D., Kostić M., Marković T., Milanović S. (2008). Potential of essential oils from anise, dill and fennel seeds for the gypsy moth control. Plants.

[57] Fujita K.I., Kubo I. (2004). Potentiation of fungicidal activities of trans-anethole against *Saccharomyces cerevisiae* under hypoxic conditions. J. Biosci. Bioeng..

[58] Huang Y., Zhao J., Zhou L., Wang J., Gong Y., Chen X., Guo Z., Wang Q., Jiang W. (2010). Antifungal activity of the essential oil of *Illicium verum* fruit and its main component trans-anethole. Molecules.

[59] Medeiros M.A.A., Alves M.S., Santos B., Silva E.V.A., Araújo F.S.M., Bezerra M.M.S.L., Silva P.O.A., Rêgo V.G.S., Pessôa H.L.F., Oliveira-Filho A.A. (2023). Evaluation of the antibacterial activity of trans-anethole against *Enterococcus cloacae* and *Enterococcus faecalis* strains of food origin. Braz. J. Biol..

[60] Bonvehí S., Coll F.V., Martínez J.A.R. (2015). Residues of essential oils in honey after treatments to control Varroa destructor. J. Essent. Oil Res..

[61] Bava R., Castagna F., Palma E., Marrelli M., Conforti F., Musolino V., Carresi C., Lupia C., Ceniti C., Tilocca B. (2023). Essential oils for a sustainable control of honeybee varroosis. Vet. Sci..

[62] Dwivedy A.K., Kumar M., Upadhyay N., Prakash B., Dubey N.K. (2016). Plant essential oils against food borne fungi and mycotoxins. Curr. Opin. Food Sci..

